# *Zbtb46^T11A^* Mutation Is Associated with Enhanced Influenza Vaccine Immunogenicity and Altered cDC1 Proportions in Mice

**DOI:** 10.3390/biology15171558

**Published:** 2026-09-06

**Authors:** Yifan Zhao, Yuxuan Lei, Qiuyi Xu, Shumiao Zhang, Qian Xie, Lifang Yuan, Ruiqi Liang, Simin Wen, Yuelong Shu

**Affiliations:** 1 School of Public Health (Shenzhen), Sun Yat-sen University, Shenzhen 518107, China; 2 Institute of Pathogen Biology, Chinese Academy of Medical Sciences & Peking Union Medical College, Beijing 102629, China; 3 Department of Hospital Administration, Sun Yat-sen University, Guangzhou 510275, China; 4 Guangzhou First People’s Hospital, Guangzhou 510180, China

**Keywords:** Zbtb46, influenza, vaccine, cDC1, immunogenicity

## Abstract

**Background:** Influenza remains a significant global public health threat, causing substantial morbidity and mortality worldwide. While vaccination serves as the best preventive strategy, considerable interindividual variability in vaccine-induced immune responses persists. The *ZBTB46* rs2281929 polymorphism (c.A31G; p.T11A; ACG>GCG), which corresponds to the evolutionarily conserved mouse mutation *Zbtb46^T11A^* (c.A31G; ACT>GCT), has been associated with enhanced antibody responses to influenza vaccination in humans, though its functional mechanisms remain unknown. This study aimed to investigate how this mutation affects influenza vaccine immunogenicity using a knock-in mouse model. **Methods:** A *Zbtb46^T11A^* knock-in mouse model was generated using CRISPR/Cas9 technology. Homozygous (HO) and wild-type (WT) mice were immunized with a quadrivalent influenza vaccine in a prime-boost regimen. Humoral immune responses were assessed by Enzyme-linked immunosorbent assay, hemagglutination inhibition (HI), and microneutralization (MN) assays. Antibody-secreting cells (ASCs) were quantified by Enzyme-linked immunospot assays. Germinal center B cells, plasma cells, plasmablast cells, conventional dendritic cell (cDC) subsets, and T helper (Th) cells were analyzed by flow cytometry. Statistical comparisons were performed using a two-sample *t*-test. **Results:** The *Zbtb46^T11A^* mutation did not alter Zbtb46 protein expression or its abundance in cDCs. Following vaccination, HO mice exhibited significantly enhanced humoral responses, including higher HA-specific IgG titers, HI and MN antibody levels, and increased numbers of ASCs. Flow cytometry revealed elevated proportions of germinal center B cells and plasma cells in HO mice. Furthermore, HO mice showed a selective expansion of type 1 cDCs (cDC1s) and a concomitant increase in Th1 cell frequencies and IFN-γ-secreting cells, while cDC2 proportions and Th2 responses remained unchanged. **Conclusions:** The *Zbtb46^T11A^* mutation is associated with enhanced influenza vaccine immunogenicity, concomitant with increased cDC1 proportions, Th1 polarization, and germinal center-dependent humoral immunity. These observed associations suggest a candidate mechanism whereby *Zbtb46* modulation may shape adaptive immunity, though further functional studies are required to establish causality. These findings provide insights into host genetic variation in vaccine responsiveness and may inform personalized vaccination strategies.

## Simple Summary

Flu vaccines do not protect everyone equally, and scientists are still trying to understand why some people respond better than others. In this study, we asked whether small genetic differences can affect how the body responds to vaccination. We used a specially bred mouse model that carries a specific genetic change to study immune responses after flu vaccination. We found that mice with this change developed stronger immune responses after vaccination, including higher levels of antibodies against influenza virus. These mice also showed enhanced activity of immune cells involved in organizing and coordinating immune responses. In addition, a group of immune cells important for initiating antiviral immune responses was increased. Overall, our results show that natural genetic differences can influence vaccine-induced immune responses by affecting immune cell composition and function. This may help explain why individuals respond differently to vaccines and provide insights into the role of host genetic variation in vaccine responsiveness.

## 1. Introduction

Influenza poses a significant global public health challenge, severely threatening human health. Vaccination remains the cornerstone of global public health strategies for mitigating both seasonal and pandemic influenza outbreaks [1]. However, substantial interindividual variability in vaccine-induced antibody responses presents a critical challenge, with genetic factors constituting one of the key contributors to this heterogeneity [2]. Although prior studies have identified several genetic loci associated with influenza vaccine immunogenicity [3,4,5], the mechanistic basis of these genetic variations, especially those related to genes regulating immune cell kinetics, remains poorly understood.

*ZBTB46*, located on human chromosome 20, is a transcription factor containing both BTB/POZ and zinc finger (ZF) domains. The C-terminal ZF domain (C2H2) of ZBTB46 mediates DNA binding, while the N-terminal BTB/POZ domain mediates protein–protein interactions. Transcriptional regulation is accomplished by sequence-specific binding of the ZF domain to regulatory regions in target genes, coupled with the recruitment of co-factors involved in chromatin remodeling and transcriptional silencing/activation [6].

In our previous two-stage genetic association analysis (based on a genome-wide association study; GWAS), we found that in the Chinese Han population, after adjusting for sex and age, the TC+CC genotype of *ZBTB46* rs2281929 was associated with a lower risk of low vaccine responsiveness compared to the TT genotype. Furthermore, individuals with the TC+CC genotype exhibited significantly higher antibody fold increases against H1N1, H3N2, and B influenza strains [7]. The rs2281929 polymorphism (c.A31G; p.T11A) represents a missense mutation located in exon 2 of the *ZBTB46* gene, which encodes the BTB/POZ domain. At the coding-sequence level, this variant is an A-to-G substitution at nucleotide 31 (ACG>GCG), which is annotated as a T-to-C polymorphism on the reverse genomic strand (genotypes TT/TC/CC); at the protein level, it results in a threonine-to-alanine substitution at residue 11 (p.T11A). To date, no studies have investigated the association between the rs2281929 locus and influenza vaccine immunogenicity.

Building upon these preliminary findings, the current study established a mouse model harboring a targeted mutation at the *ZBTB46* rs2281929 locus to validate the biological function impact of this single nucleotide polymorphism (SNP) on influenza vaccination immunogenicity and elucidate the potential mechanisms underlying this genetic regulation. This study contributes to the precise identification of high-risk populations with low immune responsiveness to influenza vaccines, optimizes personalized vaccination strategies, and offers insights for the development of novel influenza vaccine adjuvants.

## 2. Materials and Methods

### 2.1. Generation of the Zbtb46 Knock-In Mouse Model

The human *ZBTB46* rs2281929 (c.A31G; p.T11A; ACG>GCG) corresponds to an evolutionarily conserved murine locus (*Zbtb46* c.A31G; p.T11A; ACT>GCT). Using CRISPR/Cas9-mediated homology-directed repair technology, we generated a knock-in mouse model carrying the *Zbtb46^T11A^* point mutation on a C57BL/6J genetic background through Shanghai Model Organisms Center, Inc. (Shanghai, China). The procedure was as follows: Cas9 mRNA and single-guide RNA (sgRNA) were synthesized by in vitro transcription, with a single-stranded oligodeoxynucleotide (ssODN) donor template chemically synthesized. These components (Cas9 mRNA, sgRNA, ssODN) were microinjected into fertilized oocytes of C57BL/6J mice to generate F0 founders. Founder mice harboring the target mutation (confirmed by PCR and sequencing) were subsequently bred with wild-type C57BL/6J mice to produce heterozygous F1 progeny. The schematic diagram of the construction of the mouse model is shown in Supplementary Figure S1A. The animal experimental protocols were approved by the Institutional Animal Care and Use Committee (IACUC) of Sun Yat-sen University (Approval No. SYSU-IACUC-2024-001602). All procedures were conducted in compliance with both institutional regulations and national animal welfare guidelines.

### 2.2. Genotyping and Western Blotting

Genomic DNA was extracted from mouse tail snips using the One Step Mouse Genotyping Kit (Vazyme Biotech Co., Ltd., Nanjing, China). Primers were designed using the NCBI Primer-BLAST tool (https://www.ncbi.nlm.nih.gov/tools/primer-blast/, accessed on 1 July 2026) based on the nucleotide sequences flanking the target site and synthesized by Sangon Biotech (Shanghai, China) Co., Ltd. PCR amplification was performed with forward (5′-CTGGTGCCCACCTGTCATTT-3′) and reverse (5′-GTACCTGGCAGTAGAGCGTC-3′) primers, generating a single 469 bp product across all genotypes (Supplementary Figure S1B). Sequencing of the purified PCR fragments was conducted, and nucleotide sequences were aligned to the reference to confirm genotypes. As shown in Supplementary Figure S1C, homozygous (HO), heterozygous (HE), and wild-type (WT) mice were identified based on the c.A31G substitution (A → G at position 31). The BTBD4 (C-5) antibody (sc-390260, Santa Cruz Biotechnology, Dallas, TX, USA) was employed for Western blotting (WB) to assess the impact of the mutation on Zbtb46 protein expression levels.

### 2.3. Mouse Immunization Protocol

Female C57BL/6J mice (6–10 weeks) harboring a homozygous *Zbtb46^T11A^* point mutation and WT controls were used in this study. Only female mice were included to eliminate confounding effects of sex hormones and estrous cycle variability on immune responses, thereby reducing inter-individual variability and enhancing statistical power, consistent with common practice in mouse models of influenza vaccination. Mice were administered 50 μL of quadrivalent influenza vaccine (QIV) via intraperitoneal injection, with each vaccine component containing 1.5 μg hemagglutinin (HA) per strain. QIV was donated by SINOVAC BIOTECH (SINOVAC, Beijing, China), and its main components include A/Victoria/2570/2019 (H1N1) pdm09-like virus, A/Cambodia/e0826360/2020 (H3N2)-like virus, B/Washington/02/2019 (B/Victoria lineage)-like virus, and B/Phuket/3073/2013 (B/Yamagata lineage)-like virus. In accordance with the principle of fractionated dosing regimens for small animals, all subjects received two QIV immunizations separated by a 14-day interval. Experimental endpoints were established at six time points: day 7 and day 14 post-primary immunization; and day 17, day 21, day 28, and day 42 post-boost immunization (i.e., 3, 7, 14, and 28 days after the boost, respectively) (Supplementary Figure S2A).

### 2.4. Hemagglutination Inhibition and Microneutralization Assays

Hemagglutination inhibition (HI) and microneutralization (MN) assays were performed against the influenza vaccine strains according to the Manual for the laboratory diagnosis and virological surveillance of influenza [8] by the World Health Organization.

### 2.5. Enzyme-Linked Immunosorbent Assay

Flat-bottom clear plates were coated with 2 μg HA protein/well in 100 μL 50 mM Tris-HCl overnight at 4 °C. After blocking with 1% milk powder/PBS (1 h), serially diluted sera (10^−2^~10^−5^) were incubated for 2 h at room temperature (RT). Following PBS-T washes, plates were incubated with HRP-conjugated anti-mouse IgG (Proteintech, Rosemont, IL, USA, 1:1000, 1 h, 4 °C), developed with TMB substrate (Solarbio, Beijing, China, 100 μL/well), and reactions were terminated after 10 min with 1 M HCl. Absorbance (A450–A630) was measured using a ThermoFisher (Waltham, MA, USA) ELISA reader. Inter-step washes employed PBS containing 0.05% Tween-20. Ten-fold diluted positive controls on each plate enabled inter-plate normalization, while serum dilution series-derived area-under-curve (AUC) calculations determined HA-specific IgG titers.

### 2.6. Enzyme-Linked Immunospot Assay

Prior to spleen extraction, mice were euthanized in accordance with the American Veterinary Medical Association (AVMA) Guidelines for the Euthanasia of Animals and the NC3Rs guidelines on humane endpoints by intraperitoneal injection of an overdose of pentobarbital sodium followed by cervical dislocation. Serial two-fold dilutions of splenocytes were performed during assay optimization to ensure that spot counts fell within the linear detection range; 2 × 10^5^ cells per well was selected as the optimal input cell number based on these titrations. Enzyme-linked Immunospot (ELISpot) plates (96-well) were coated with 100 μL/well of QIV solution diluted 1:20 in PBS. Following overnight incubation, plates were blocked, and serially diluted mouse splenic single-cell suspensions (2 × 10^5^ cells per well) were added. After 18–20 h of incubation at 37 °C, cells were discarded. Subsequently, plates were incubated with primary antibody (Biotinylated Goat Anti-Mouse IgG) for 2 h at 37 °C. After removal of the antibody solution and washing, Streptavidin-ALP for ELISpot was added and incubated for 1 h at 37 °C. Following another washing step, filtered chromogenic substrate (BCIP/NBT) was added and developed until distinct spots emerged. Finally, plates were washed, air-dried, and spot enumeration was performed using an ELISpot Reader System.

### 2.7. Flow Cytometry Data Acquisition and Analysis

Single-cell suspensions prepared from murine spleens underwent sequential processing including Fc receptor blocking, viability staining with a fixable dead-cell discriminator dye, and surface staining with fluorochrome-conjugated antibodies; stained cells were resuspended in flow cytometry buffer, transferred to polystyrene tubes, and subjected to flow cytometric acquisition using the following fluorochrome-conjugated antibodies: anti-mouse CD3 antibody (Biolegend, clone 17A2), anti-mouse/human CD45R/B220 antibody (Biolegend, clone RA3-6B2), anti-mouse CD95 (Fas) antibody (Biolegend, clone SA367H8), anti-mouse/human GL7 antigen–antibody (Biolegend, clone GL7), anti-mouse CD138 (Syndecan-1) antibody (Biolegend, clone 281-2), anti-mouse CD11c antibody (Biolegend, clone N418), anti-mouse I-A/I-E antibody (Biolegend, clone M5/114.15.2), anti-mouse/human CD11b antibody (Biolegend, clone M1/70), anti-mouse/rat XCR1 antibody (Biolegend, clone ZET), anti-mouse CD4 antibody (Biolegend, clone GK1.5), anti-mouse CD194 (CCR4) antibody (Biolegend, clone 2G12), anti-mouse CD183 (CXCR3) antibody (Biolegend, clone CXCR3-173), anti-mouse CD196 (CCR6) antibody (Biolegend, clone 29-2L17) and anti-mouse Zbtb46 antibody (BD, clone U4-1374). All flow cytometry data were acquired using a Cytoflex S flow cytometer (Beckman Coulter, Brea, CA, USA) and analyzed with FlowJo software (v10.8.1, BD, Ashland, OR, USA). Gating strategies are shown in Supplementary Figure S2B–D.

### 2.8. Statistical Analysis

Continuous variables are expressed as mean ± standard deviation. HI and MN antibody titers were log_2_-transformed prior to statistical comparison. Differences in immunological parameters between HO mice and WT mice were compared using the two-sample *t*-test. Flow cytometry analysis and graph generation were performed using FlowJo software (v10.8.1, BD, Ashland, OR, USA). Statistical analysis and graph plotting were conducted using GraphPad Prism (v8.0). A *p*-value less than 0.05 is considered statistically significant, denoted by *; the *p*-value less than 0.01 is denoted by **; and the *p*-value less than 0.001 is denoted by ***.

## 3. Results

### 3.1. The Zbtb46^T11A^ Mutation Does Not Affect Zbtb46 Protein Expression or Its Abundance in cDCs

**Figure 1:**
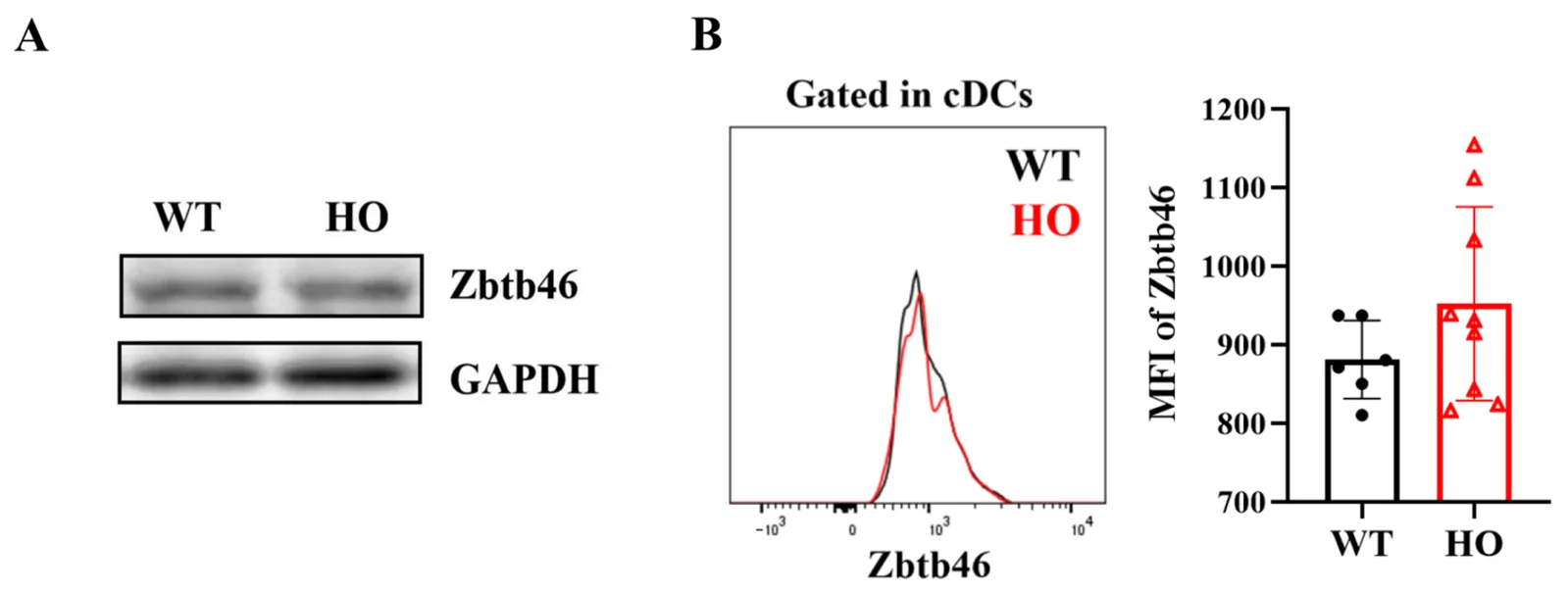
The *Zbtb46^T11A^* mutation does not affect the expression of the Zbtb46 protein or its abundance in cDCs. (**A**) Western blot detection of Zbtb46 protein expression in HO and WT mice. Full-length blots/gels are presented in Supplementary Figure S3. (**B**) Zbtb46 expression levels in cDCs of different genotypes of mice (*n* = 6 for WT, *n* = 10 for HO). Abbreviations: HO, homozygous; HE, heterozygous; WT, wild-type; cDC, conventional dendritic cell; MFI, mean fluorescence intensity. Data are presented as mean ± standard deviation (SD).

We generated the knock-in mouse model using CRISPR/Cas9 technology (Supplementary Figure S1A) and verified the genotypes by sequencing (Supplementary Figure S1B,C). WT, HE, and HO mice showed AA, AG, and GG genotypes at nucleotide position 31, respectively. Through Western blotting and flow cytometry analysis, we confirmed that the mutation does not affect Zbtb46 protein expression (Figure 1A) or its abundance in cDCs (Figure 1B).

### 3.2. Enhanced Humoral Responses in HO Mice Post-Vaccination

**Figure 2:**
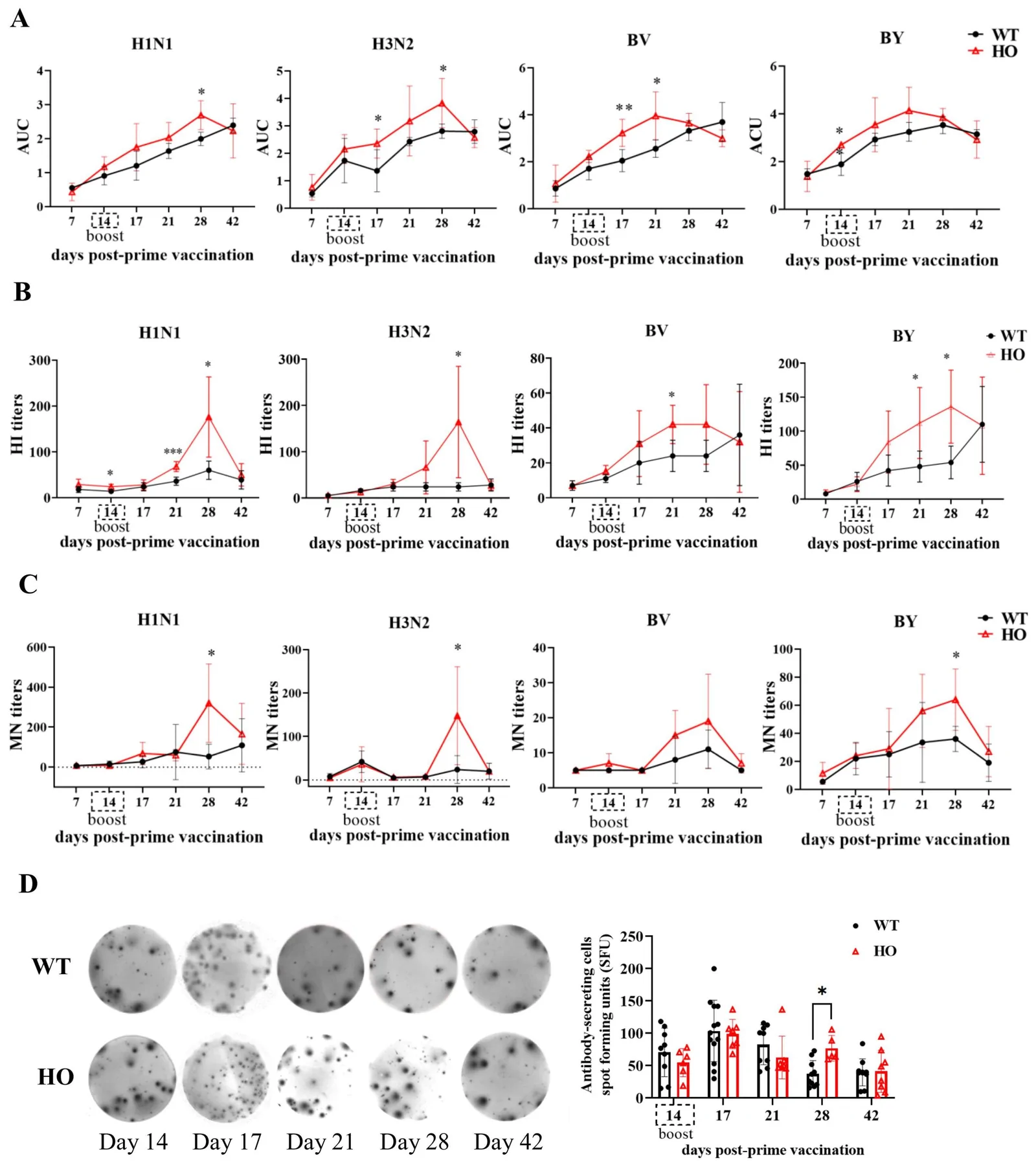
HO mice exhibit enhanced humoral immune responses following influenza vaccination. (**A**–**C**) HO mice showed elevated levels of HA-specific IgG antibodies, HI antibodies, and MN antibodies relative to WT mice after vaccination. (**D**) Total antibody-secreting cells (ASCs) were significantly higher in HO mice compared to WT mice at day 28 post-vaccination. * *p* < 0.05, ** *p* < 0.01, *** *p* < 0.001. Abbreviations: HO, homozygous; WT, wild-type; AUC, area under the curve; HI, hemagglutination inhibition; MN, microneutralization. Data are presented as mean ± standard deviation (SD).

To assess the effect of the *Zbtb46^T11A^* mutation on influenza vaccine immunogenicity, we immunized mice with a commercial quadrivalent split-vaccine using a prime-boost strategy (Supplementary Figure S2A). Humoral immune responses were comprehensively evaluated using serum and splenocytes from different genotypes. The enzyme-linked immunosorbent assay (ELISA), HI, and MN assay results showed that HO mice exhibited enhanced serum antibody responses, including higher HA-specific IgG titers (Figure 2A), elevated HI antibody levels (Figure 2B), and increased neutralizing antibody activity (Figure 2C). Consistent with this, the ELISpot assay demonstrated a significant increase in antibody-secreting cells (ASCs) in HO mice compared to WT controls at day 28 post-vaccination (Figure 2D).

**Figure 3:**
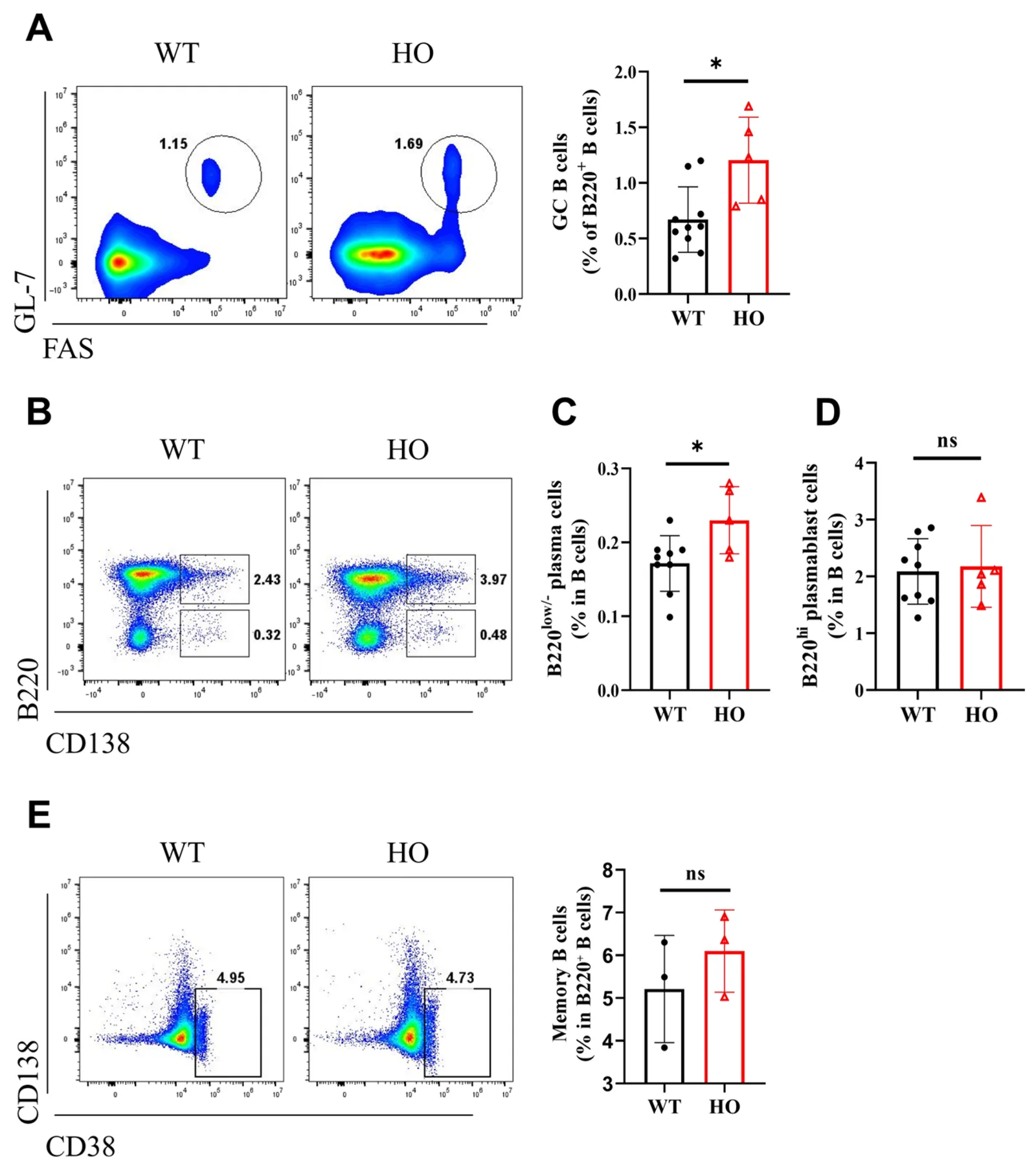
The proportions of GC B cells and CD138^+^ B cells in HO and WT mice at day 28 post-vaccination. (**A**) HO mice showed significantly higher proportions of GC B cells (*n* = 10 for WT, *n* = 5 for HO). (**B**,**C**) HO mice showed significantly higher proportions of B220^low/−^ plasma cells compared to WT mice (*n* = 9 for WT, *n* = 5 for HO), while (**B**,**D**) no significant difference was observed in B220hi plasmablast cells (*n* = 9 for WT, *n* = 5 for HO). (**E**) Representative flow cytometry plots and quantification showed no significant difference in the proportions of memory B cells (% in B220^+^ B cells) between HO and WT mice (*n* = 3 for WT, *n* = 3 for HO). * *p* < 0.05. Abbreviations: HO, homozygous; WT, wild-type; GC, germinal center. Data are presented as mean ± standard deviation (SD). ns: no significant.

Flow cytometry analyses of splenocytes collected at 28 days post-vaccination revealed that HO mice exhibited significantly higher proportions of germinal center (GC) B cells (Figure 3A) and B220^low/−^ plasma cells (Figure 3B,C) compared to WT mice. However, no significant difference was observed in the proportion of B220^hi^ plasmablast cells among B cells between groups (Figure 3B,D). Likewise, representative flow cytometry plots and quantitative analysis showed comparable proportions of memory B cells between HO and WT mice (Figure 3E).

### 3.3. Higher cDC1 and Th1 Proportions in HO Mice Compared to WT Mice Following Influenza Vaccination

**Figure 4:**
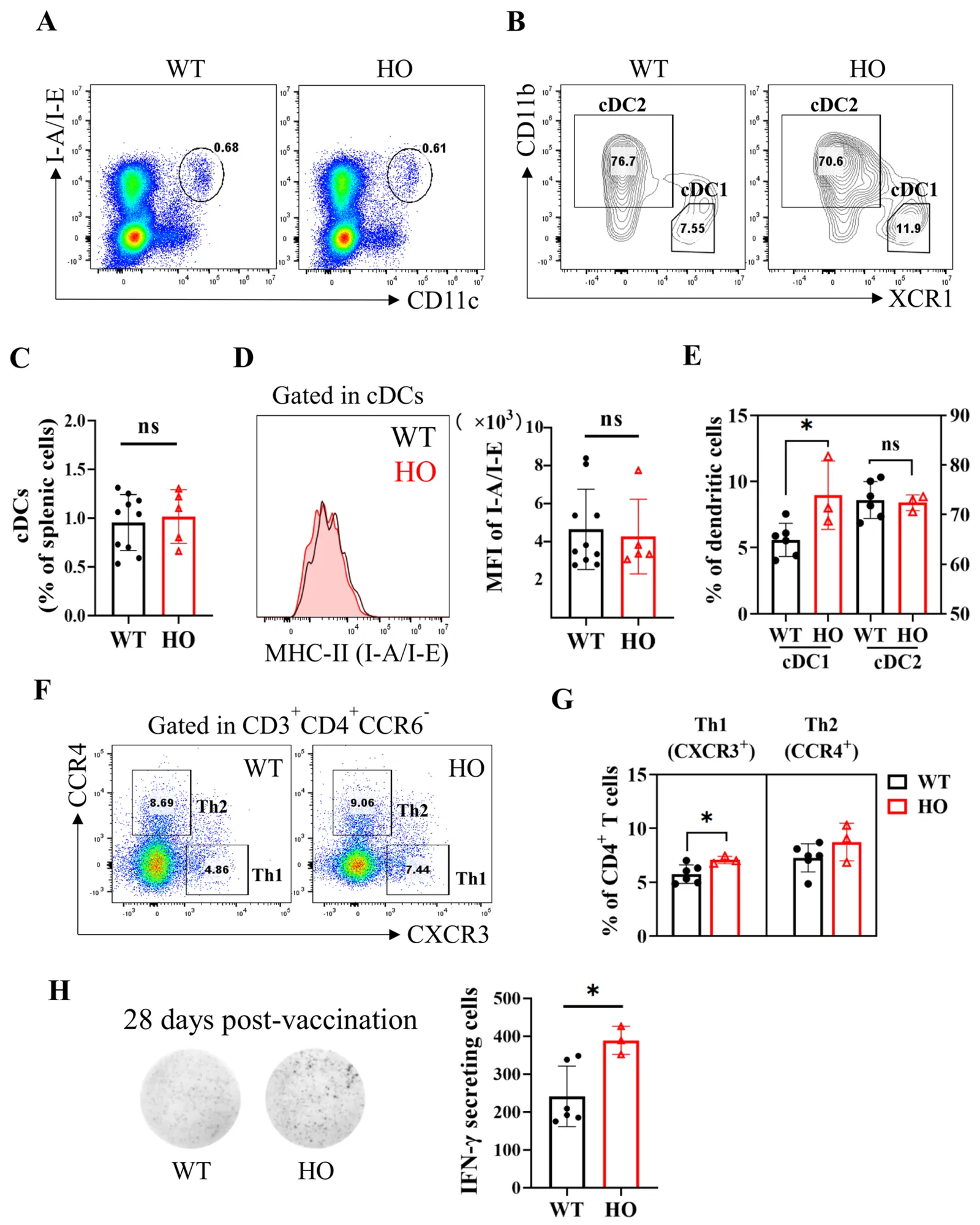
Enhanced cDC1 and Th1 responses in HO mice at day 28 post-vaccination. (**A**) Flow cytometry was performed to analyze splenic cDC populations across genotypes. (**B**) Representative flow plots show the proportions of cDC1 (XCR1+) and cDC2 (CD11b+) subsets in different genotypes. (**C**) Total splenic cDC proportions were comparable between HO and WT mice (*n* = 10 for WT, *n* = 5 for HO). (**D**) No significant differences were observed in MHC-II expression in splenic cDCs across genotypes post-vaccination (*n* = 10 for WT, *n* = 5 for HO). (**E**) HO mice exhibited an increased proportion of cDC1 with an unchanged percentage of cDC2 post-vaccination (*n* = 6 for WT, *n* = 3 for HO for both cDC1 and cDC2 subsets). (**F**) Changes in Th1 (CXCR3+) and Th2 (CCR4+) cell proportions post-vaccination are shown. (**G**) The proportion of Th1 cells was significantly higher in HO mice compared to WT mice, while the proportion of Th2 cells showed no statistically significant difference between the two groups (*n* = 6 for WT, *n* = 3 for HO for both Th1 and Th2 subsets). (**H**) The proportion of IFN-γ-secreting cells was higher in HO mice than in WT mice (*n* = 6 for WT, *n* = 3 for HO). * *p* < 0.05. Abbreviations: HO, homozygous; WT, wild-type; cDC, conventional dendritic cell; MHC, major histocompatibility complex; MFI, mean fluorescence intensity; Th, T helper; IFN-γ, interferon-γ. Data are presented as mean ± SD. ns: no significant.

As Zbtb46 serves as a specific marker for conventional dendritic cells (cDCs) in the spleen, we investigated how the mutation affects the total cDCs (Figure 4A) and the distribution of cDC subsets (Figure 4B) following vaccination. At 28 days post-vaccination, we observed that both the total cDCs (Figure 4A,C) and MHC II expression levels in cDCs (Figure 4D) showed no significant differences between HO and WT mice. HO mice displayed a significantly increased proportion of cDC1s within the cDCs compared to WT mice, whereas the distribution of cDC2s remained comparable between the two genotypes (Figure 4B,E), indicating the observed effect was specific to the cDC1 subpopulation.

The two major cDC subsets, cDC1 (XCR1^+^) and cDC2 (CD11b^+^), are known to preferentially induce T helper 1 (Th1) and Th2 polarization, respectively [9,10]. Consistent with the observed expansion of cDC1s in HO mice post-vaccination, flow cytometric analysis showed a significant increase in Th1 responses in HO mice compared to WT controls (Figure 4F,G). However, the mutation did not significantly alter Th2 cell frequencies. This Th1-skewed immune polarization was further validated by ELISpot assays, which revealed a marked increase in IFN-γ-secreting cells in HO mice (Figure 4H). Considering the essential function of cDCs in supporting Tfh cell generation and differentiation during vaccine-induced immune responses, we further analyzed Tfh cell proportions in CD4^+^CD44^+^ effector T cells. Flow cytometry quantification demonstrated no significant difference in Tfh cell frequencies between HO and WT mice at day 28 post-vaccination (*n* = 9 for WT, *n* = 3 for HO; Student’s *t*-test) (Figure S2E).

## 4. Discussion

This study is the first to investigate the impact of heritable variations in *Zbtb46* on vaccine-induced adaptive immune responses. We observed that the *Zbtb46^T11A^* mutation is associated with higher levels of HA-specific serum IgG antibodies, HI antibodies, and MN antibodies after influenza vaccination. This mutation was associated with increased proportions of GC B cells, plasma cells, cDC1 subsets, and Th1-associated immune responses, and was accompanied by higher levels of antibody-secreting cells and IFN-γ-secreting cells. Our preliminary population-based genetic association analyses also revealed that individuals carrying this mutation exhibit significantly higher antibody levels following influenza vaccination [7]. Together, these findings suggest that the *Zbtb46^T11A^* variant may influence influenza vaccine-induced immune responses through modulation of multiple immune cell populations. However, further functional studies are required to determine the precise molecular and cellular mechanisms underlying these associations.

While annual influenza vaccination effectively reduces epidemic spread and disease burden, CDC data (2009–2025) show suboptimal population protection (mean VE = 42.9%) [11]. Due to this subpar effectiveness, influenza continues to pose a serious threat to the health and lives of high-risk groups, including pregnant women, individuals with underlying medical conditions, young children, and the elderly. This limited protection stems from variable immunogenicity influenced by vaccine design, administration history, and host factors [12,13,14,15]. Our prior population-based study linked the *ZBTB46* rs2281929 variant to enhanced vaccine responses, prompting this mechanistic investigation [7].

Zbtb46 (also known as zDC) is a cDC-specific transcription factor absent in macrophages and monocytes [16,17,18]. Meredith et al. employed ChIP-seq analysis to identify Zbtb46 target genes, revealing its direct binding to multiple cDC-associated loci, including MHC-II genes, thus establishing its role as a negative regulator of cDC gene expression [19]. Using *Zbtb46*-knockout (KO) mice, flow cytometric analysis demonstrated that while Zbtb46 deletion preserves total cDC numbers, it alters splenic CD4^+^ (cDC2) and CD8^+^ (cDC1) cDC ratios. In this study, we found that the *Zbtb46^T11A^* mutation similarly preserved total splenic cDC numbers while being associated with an increased proportion of the cDC1 (CD8^+^ cDC) [9] subset. However, unlike previous knockout studies, this point mutation does not alter MHC-II expression levels or affect cDC2 (CD4^+^ cDC) distribution. These findings suggest that selective modulation of cDC1 population dynamics may represent a candidate mechanism underlying the enhanced influenza vaccine-induced immune response observed in *Zbtb46^T11A^* mice.

cDCs are professional antigen-presenting cells (APCs) that play pivotal roles in initiating and orchestrating immune responses. cDCs are generally classified into two major subsets: conventional DC1 (cDC1) and conventional DC2 (cDC2) [9,10,18]. Our flow cytometric analysis revealed that the *Zbtb46^T11A^* variant was associated with an increased frequency of cDC1s within splenic DC populations while maintaining unchanged cDC2 proportions. cDC1s are particularly specialized for cross-presenting cell-associated antigens, making them crucial for priming CD8^+^ T cell responses against viruses and tumors. Notably, emerging evidence indicates that cDC1s also contribute to CD4^+^ T cell priming against tumor antigens [20] and are essential for both CD4^+^ T cell intestinal homing and infection site IL-12 production, thereby promoting localized IFN-γ responses [21]. Consistent with these functions, we found that HO mice with elevated cDC1 frequencies also exhibited increased splenic Th1-associated immune responses, including higher proportions of Th1 cells and greater numbers of IFN-γ-secreting cells compared with WT controls.

Previous studies have indicated that targeting antigen to cDC1s may also result in improved humoral responses [22,23]. Vaccine molecules targeting XCR1 on cross-presenting DCs elicit robust CD8^+^ T-cell responses along with Th1-polarized CD4^+^ T-cell immunity and selective IgG2a antibody production against influenza virus [24]. Similarly, XCL1-fusion vaccines generate IgG2a/IgG2b-dominated antibody responses and promote rapid Th1 cell polarization both in vitro and in vivo [25]. Thus, the observed increase in cDC1 frequency and subsequent Th1 cell polarization may contribute to the enhanced influenza vaccine immunogenicity in HO mice. Notably, however, no significant differences were observed in splenic T follicular helper (Tfh) cell frequencies or memory B-cell proportions between genotypes, suggesting that the enhanced humoral immunogenicity is likely driven by the increased germinal center B-cell and plasma cell populations rather than by expanded Tfh or memory B-cell compartments.

The limitations of this study mainly include the following aspects: First, both the serological and cellular assays in this study only compared homozygous mutant mice with wild-type mice, without analyzing heterozygous mice. This may lead to an incomplete understanding of the dosage effect of the gene and potential biological compensatory mechanisms. Given that the rs2281929 variant is predominantly carried in the heterozygous state in human populations, future studies including heterozygous mice will be essential to determine whether the observed immune effects exhibit a gene dosage effect and to improve the clinical translatability of these findings. Second, although the ZBTB46 protein abundance was unchanged in mutant mice, the potential effects of the T11A substitution on protein function remain unclear. Further studies investigating DNA-binding activity, transcriptional regulation, protein stability, cellular localization, and protein–protein interactions will be required to clarify the molecular consequences of this missense mutation. Third, functional experiments directly evaluating cDC1 antigen presentation capacity, T-cell priming ability, and cytokine production were not performed in this study. Therefore, the contribution of cDC1 functional changes to the observed immune phenotypes requires further investigation. Furthermore, the statistical analyses in this study relied primarily on two-sample *t*-tests for pairwise comparisons at individual time points, and formal normality testing (e.g., Shapiro–Wilk test) was not performed. For longitudinal antibody kinetics data, repeated-measures ANOVA or mixed-effects models would provide a more robust evaluation of genotype-by-time interactions, and these approaches will be incorporated in future studies with larger sample sizes. Fourth, this study focused on vaccine-induced immunogenicity and did not include influenza virus challenge experiments; therefore, whether the *Zbtb46^T11A^* variant provides enhanced protection against influenza infection remains to be determined. In addition, antibody analysis was primarily based on HA-specific IgG levels, and future studies evaluating IgG subclasses will provide further insights into antibody polarization. Finally, only female mice were evaluated, and future studies involving both male and female mice will be necessary to determine whether the observed immune effects are sex-dependent.

## 5. Conclusions

This study establishes a functional link between the *ZBTB46* rs2281929 polymorphism and influenza vaccine responses using a precisely engineered knock-in mouse model. We show that the *Zbtb46^T11A^* variant is associated with enhanced humoral and cellular responses to influenza vaccination in mice, concomitant with altered cDC1 subset proportions and increased germinal center activity. The cDC1–Th1 axis may therefore contribute to the enhanced immunogenicity, although direct causal evidence—such as cDC1 depletion or functional antigen-presentation assays—awaits experimental validation. By bridging human GWAS data with mechanistic hypotheses in a mouse model, this work highlights Zbtb46-cDC1 modulation as a candidate pathway underlying variable vaccine responsiveness. Looking ahead, determining whether targeting this axis can improve vaccine efficacy may inform precision vaccinology and stratified vaccination strategies.

## Data Availability

The original contributions presented in this study are included in the article and Supplementary Materials. Further inquiries can be directed to the corresponding authors.

## Supplementary Materials

The following supporting information can be downloaded at: https://www.mdpi.com/article/10.3390/biology15171558/s1, Figure S1: Construction and genotype identification of *Zbtb46^T11A^* knock-in mice. (A) Schematic of targeting strategy. (B) Electrophoresis image of PCR products. (C) Alignment of nucleotide sequences among the three types of mice. Abbreviations: HO, homozygous; HE, heterozygous; WT, wild-type. Figure S2: Immunization protocol and immune cell gating strategies (A) Schematic of mouse immunization timeline. (B–D) Flow cytometry gating approaches for T cell subsets, B cell subsets (including memory B cells defined as Fas^−^ GL7^−^ CD38hi cells), and cDC subsets. Abbreviations: cDC, conventional dendritic cell; MHC, major histocompatibility complex. (E) Representative flow cytometry gating strategy and quantification for Tfh cells (% in CD4^+^CD44^+^ T cells). Abbreviations: cDC, conventional dendritic cell; MHC, major histocompatibility complex; Tfh, T follicular helper. Figure S3: Full-length blots/gels for WB determination of Zbtb46 protein expression. The cropped areas used in the main figures are indicated by boxes.
