# Supplementary material for: Zbtb46T11A Mutation Is Associated with Enhanced Influenza Vaccine Immunogenicity and Altered cDC1 Proportions in Mice
Source: Biology (Basel). 2026 Sep 6;15(17):1558. doi: 10.3390/biology15171558 (PMC13565068; doi:10.3390/biology15171558)
Supplement: Supplementary file 1 [file biology-15-01558-s001.zip › Supplementary Figure S1.pdf]

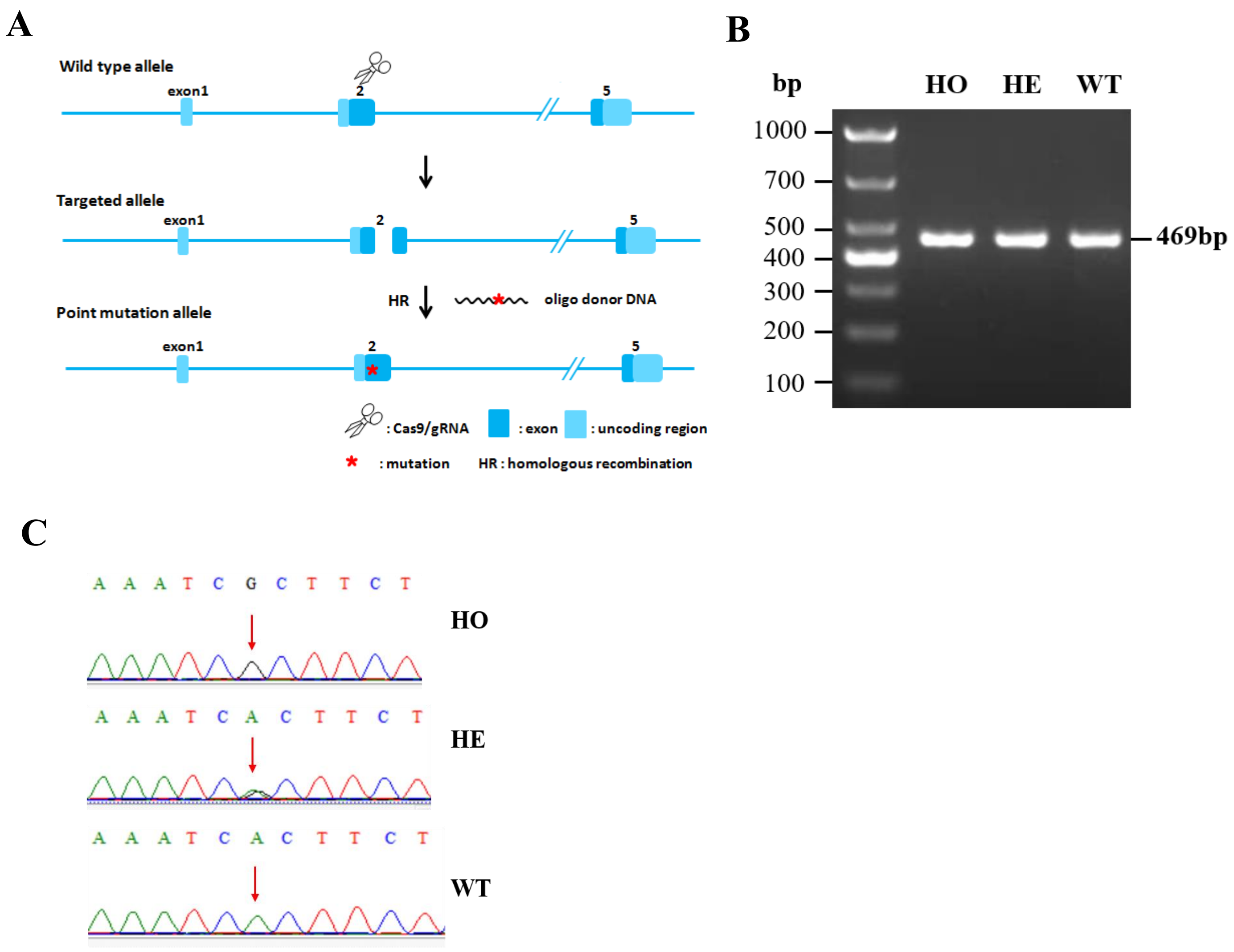


**Figure S1.** Construction and genotype identification of *Zbtb46*$^{T11A}$ knock-in mice. (A) Schematic of targeting strategy. (B) Electrophoresis image of PCR products. (C) Alignment of nucleotide sequences among the three types of mice. Abbreviations: HO, homozygous; HE, heterozygous; WT, wild-type.
