# Supplementary material for: Zbtb46T11A Mutation Is Associated with Enhanced Influenza Vaccine Immunogenicity and Altered cDC1 Proportions in Mice
Source: Biology (Basel). 2026 Sep 6;15(17):1558. doi: 10.3390/biology15171558 (PMC13565068; doi:10.3390/biology15171558)
Supplement: Supplementary file 1 [file biology-15-01558-s001.zip › Supplementary Figure S2.pdf]

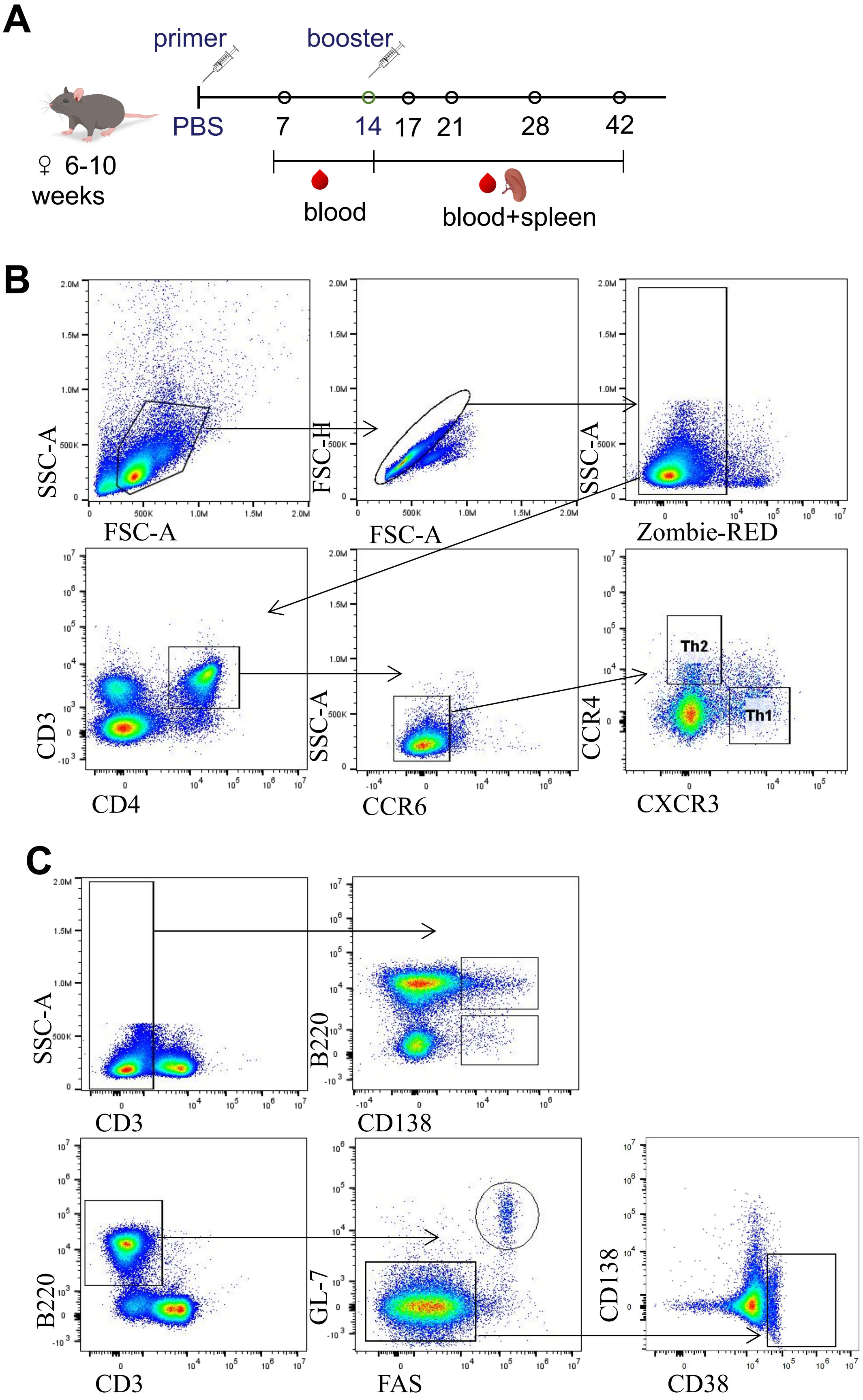
A
primer
booster
PBS
7
14
17
21
28
42
♀ 6-10 weeks
blood
blood+spleen
B
SSC-A
FSC-A
FSC-H
FSC-A
SSC-A
Zombie-RED
CD3
CD4
SSC-A
CCR6
CCR4
CXCR3
Th2
Th1
C
SSC-A
CD3
B220
CD138
B220
CD3
GL-7
FAS
CD138
CD38

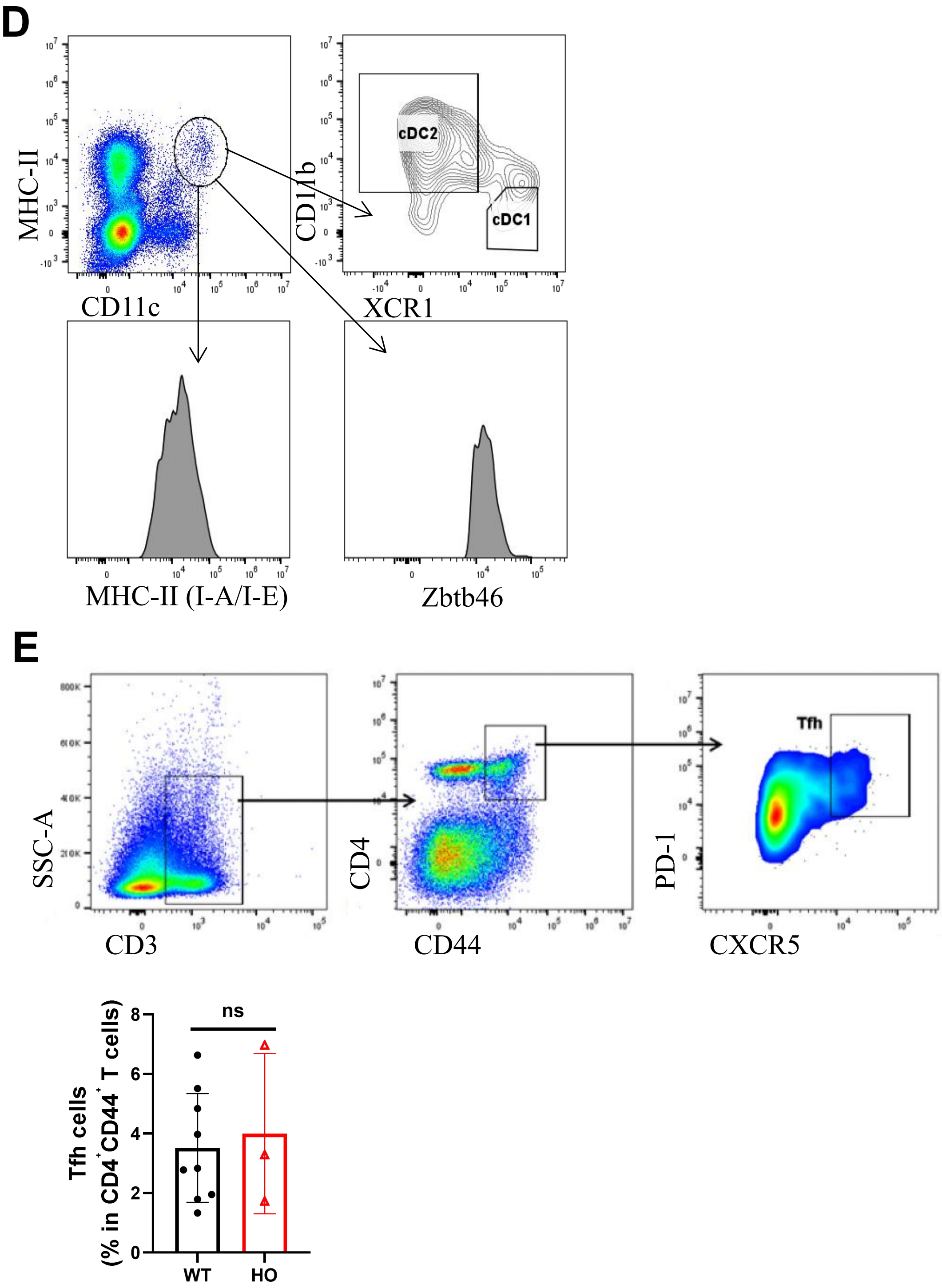

Figure S2. Immunization protocol and immune cell gating strategies (A) Schematic of mouse immunization timeline. (B-D) Flow cytometry gating approaches for T cell subsets, B cell subsets (including memory B cells defined as Fas^{-} $GL7^{-}$ $CD38^{hi}$ cells), and cDC subsets. Abbreviations: cDC, conventional dendritic cell; MHC, major histocompatibility complex. (E) Representative flow cytometry gating strategy and quantification for Tfh cells (% in $CD4^{+}CD44^{+}$ T cells). Abbreviations: cDC, conventional dendritic cell; MHC, major histocompatibility complex; Tfh, T follicular helper.
