# Supplementary material for: Zbtb46T11A Mutation Is Associated with Enhanced Influenza Vaccine Immunogenicity and Altered cDC1 Proportions in Mice
Source: Biology (Basel). 2026 Sep 6;15(17):1558. doi: 10.3390/biology15171558 (PMC13565068; doi:10.3390/biology15171558)
Supplement: Supplementary file 1 [file biology-15-01558-s001.zip › Supplementary Figure S3.pdf]

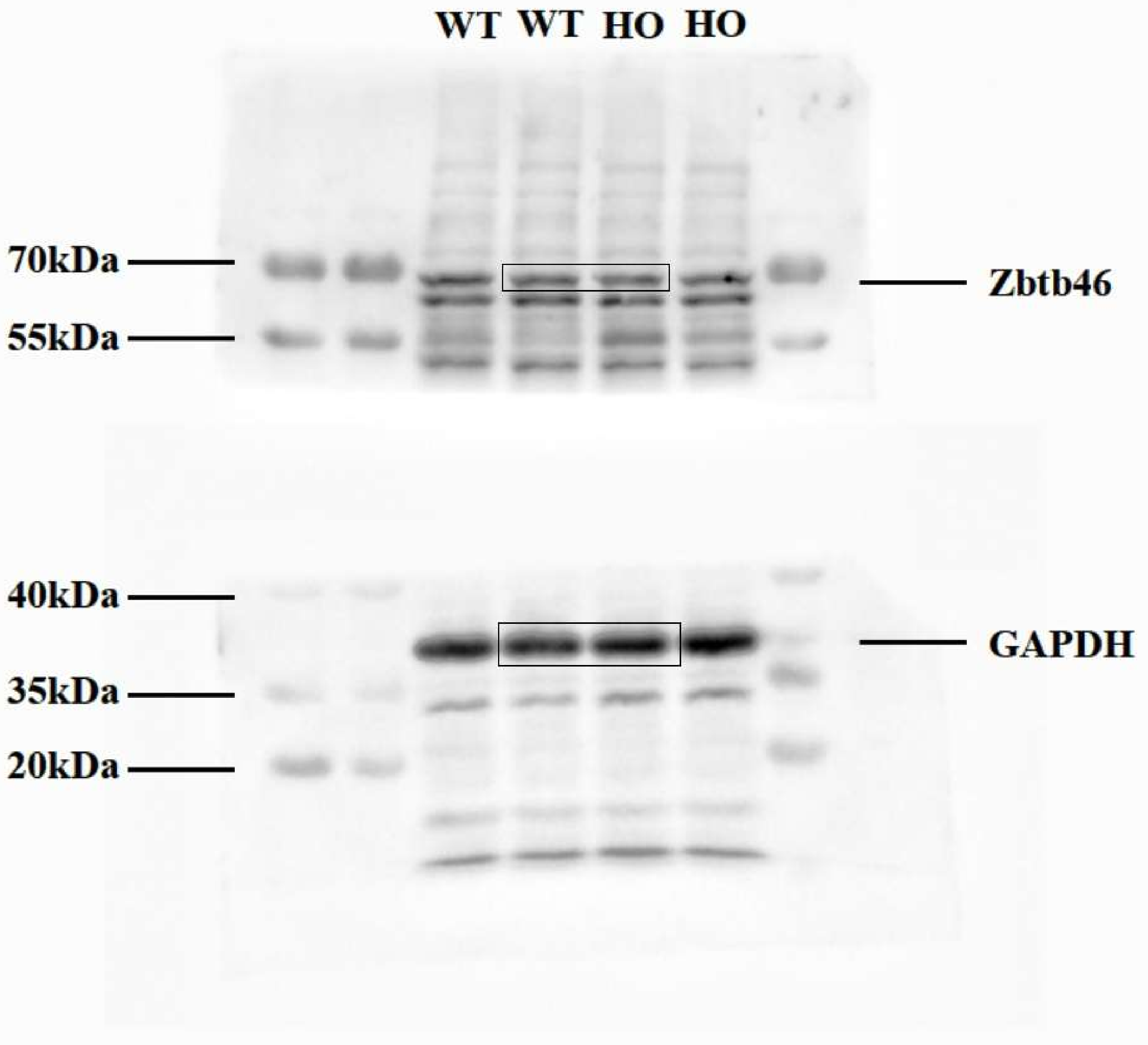
WT WT HO HO
70kDa
55kDa
Zbtb46
40kDa
35kDa
20kDa
GAPDH
